# A phase I study of a dual PI3-kinase/mTOR inhibitor BEZ235 in adult patients with relapsed or refractory acute leukemia

**DOI:** 10.1186/s40360-020-00446-x

**Published:** 2020-09-29

**Authors:** Fabian Lang, Lydia Wunderle, Susanne Badura, Eberhard Schleyer, Monika Brüggemann, Hubert Serve, Susanne Schnittger, Nicola Gökbuget, Heike Pfeifer, Sebastian Wagner, Kevin Ashelford, Gesine Bug, Oliver G. Ottmann

**Affiliations:** 1 Department of Medicine, Hematology and Oncology, Goethe University Hospital, Frankfurt, Germany; 2 Medical Clinic and Policlinic 1, Hematology and Cellular Therapy, Leipzig University Hospital, Leipzig, Germany; 3 Department of Hematology, UKSH, Campus Kiel, Kiel, Germany; 4 DKFZ Heidelberg, Heidelberg, Germany; 5 Münchner Leukämie Labor, Munich, Germany; 6 School of Medicine, Cardiff University, Cardiff, Wales UK

**Keywords:** Refractory ALL, Refractory AML, PI3K/mTor inhibition, BEZ235, Phase I clinical trial

## Abstract

**Background:**

Combined inhibition of phosphatidylinositol 3-kinase (PI3K) and the mammalian target of rapamycin (mTOR) complexes may be an efficient treatment for acute leukemia. The primary objective of this phase I single center open label study was to determine the maximum tolerated dose (MTD) and recommended phase II dose (RP2D) of the dual pan-class I PI3K and mTOR inhibitor BEZ235 in patients with advanced leukemia.

**Methods:**

Herein patients > 18 years of age who had relapsed or showed refractory leukemia were treated with BEZ235 (orally at 300–400 mg BID (cohort − 1/1)) to assess safety, tolerability, preliminary efficacy and pharmacokinetic (PK). Adverse events data and serious adverse events were analyzed and haematological and clinical biochemistry toxicities were assessed from laboratory test parameters. Response was assessed for the first time at the end of cycle 1 (day 29) and after every subsequent cycle. Pharmacokinetic and pharmacodynamic analyses of BEZ235 were also included (BEZ235 plasma levels, phosphorylation of AKT, S6 and 4EBP1). On statistics this trial is a multiple ascending dose study in which a following variant of the 3 + 3 rule (“Rolling Six”), a minimum of 6 and a maximum of 12 patients was recruited for the dose escalation and another 5 were planned for the expansion phase.

**Results:**

Twenty-four patients with ALL (*n* = 11) or AML (*n* = 12) or CML-BP (*n* = 1) were enrolled. All patients had failed one (*n* = 5) or more lines of therapy (*n* = 5) and 14 patients were in refractory / refractory relapse. No formal MTD was defined, stomatitis and gastrointestinal toxicity at 400 mg BID dose was considered incompatible with prolonged treatment. The RP2D of BEZ235 was defined as 300 mg BID. Four of 24 patients showed clinical benefit. Twenty-two of 24 patients discontinued because of progression, (median time to progression 27 days (4d-112d). There was no association between PK parameters and efficacy or tolerability.

**Conclusions:**

Combined inhibition of PI3K and mTOR inhibits a clinically meaningful driver pathway in a small subset of patients with ALL, with no benefit in patients with AML.

**Trial registration:**

ClinicalTrials.gov, identifier NCT01756118. retrospectively registered 19th December 2012, https://clinicaltrials.gov/ct2/show/NCT01756118.

## Background

The phosphatidylinositol 3-kinase (PI3K) / Akt / mammalian target of rapamycin (mTOR) signaling axis plays an important physiologic role in protein synthesis, gene transcription, cell growth and apoptosis [1, 2]. Oncogenic activation of the PI3K pathway has been shown in a variety of solid tumors and hematologic malignancies and has been linked to treatment resistance and disease progression [3–9].

In acute leukemias PI3K/Akt signaling activity was demonstrated to be correlated with an inferior prognosis via contribution to proliferation, survival and drug resistance in acute myeloid leukemia [3, 10–12], in T-cell acute lymphoblastic leukemia (T-ALL) [13] and B-cell precursor acute lymphoblastic leukemia (BCP-ALL) [14–16]. Herein in Philadelphia chromosome positive (Ph+) BCP-ALL PI3K signaling has been shown to be involved in mutation-independent resistance to ABL-directed tyrosine kinase inhibitors [14] and recent preclinical data also suggest a role in Philadelphia chromosome negative (Ph-) BCP-ALL [14, 15].

Dysregulation may occur at different levels of the PI3K/AKT/mTOR signalling cascade and involve distinct mechanisms, the prevalence of which differs depending on the disease entity. While mutations of the *PI3KCA* gene are rare in leukemia, events upstream of PI3K are commonly implicated in causing aberrant activation of this pathway, e.g. activating mutations of the receptor tyrosine kinases (RTKs), Fms-like tyrosine kinase 3 (*FLT3*) and *KIT* receptor tyrosine kinase, *BCR-ABL1* fusion gene, or activating mutations in *NRas* and *KRas* [17–20]. Inactivation of the phosphatase and tensin homolog deleted on chromosome 10 (*PTEN*), a negative regulator of the PI3K pathway, has also been observed in acute leukemias [21] as well as activation of AKT as a consequence of *NOTCH1* activating mutations [7, 22]. Notably, PI3K/Akt/mTOR network up-regulation has been detected in leukemia stem cells (LSCs) transplanted in non-obese diabetic/severe combined immunodeficiency (NOD/SCID) mice, where it exerted a powerful pro-survival effect [23, 24]. Taken together, these observations lent compelling weight to the clinical exploration of PI3K inhibitors in the therapy of acute leukemia.

Pharmacologic inhibition of PI3K signalling exerted pronounced anti-proliferative effects and induced apoptosis in pre-clinical models using leukemia cell lines or primary leukemia samples, but identified limitations of selectively targeting single nodes within the PI3K/Akt/mTOR cascade. The mTOR serine/threonine kinase belongs to two separate complexes: mTORC1 and mTORC2. The mTORC1 pathway is rapamycin sensitive and controls protein translation through the phosphorylation of 4E-BP1 in most models. Furthermore, the activity of PI3K/Akt and mTOR is closely related, as mTORC2 activates the oncogenic kinase Akt. As a consequence, specific mTORC1 inhibitors such as RAD001 acting downstream of AKT, have the disadvantage of counteracting their own effects by activating AKT via feed-back mechanisms [17, 18, 24–32]. The resulting rationale for dual inhibition of both pathways is supported by preclinical data demonstrating enhanced antileukemic activity when distal and proximal nodes of the PI3K signalling cascade were inhibited simultaneously [5, 14, 19, 25, 26, 33]. Despite this encouraging preclinical data and the unmet clinical need in patients with acute leukemia who have relapsed or are refractory to induction treatment, no clinical trials of PI3K pathway inhibitors other than the mTORC1 inhibitor RAD001 have to our knowledge been conducted in patients with acute leukemia.

BEZ235 is a potent dual pan-class I PI3K and mTOR inhibitor belonging to the class of imidazoquinoline derivatives. BEZ235 belongs to the class of pan-class I PI3K inhibitors as it binds the four isoforms of PI3K (certainly preferring the α-isoform). BEZ235 binds to Valine-882 and Serine-805 in the hinge region of the ATP-binding pocket of the p110 subunit of PI3K and to the catalytic site of mTOR which leads to inhibition of both mTORC complexes (mTORC1 and mTORC2). By this, downstream effectors of PI3k (i.e. pAKT, GSK3β, p70^S6K^ and ribosomal protein S6) are inhibited in various preclinical models like cell lines and xenografts resulting in effective inhibition of tumor proliferation and growth. This effect is independent of the genetic background in different preclinical models. However, a screen of various breast cancer cell lines showed preferential inhibition of PIK3CA mutated and ERBB2 amplified cells including the induction of cell death, although generally all of the screened cell lines showed great sensitivity for the anti-proliferative effect [34].

BEZ235 has been evaluated as single agent and in combination with trastuzumab in a phase I/Ib trial [CBEZ235A2101] in patients with advanced/metastatic solid tumors. In addition, a phase I study in Japanese patients [CBEZ235A1101], a phase I study evaluating a twice daily (BID) schedule [CBEZ235ZUS07T] and a phase Ib combination trial with paclitaxel ± trastuzumab [CBEZ235A2118] were conducted. Herein the recommended dose for phase II trials was 1000 mg/d after a total of over 178 patients being treated. Notably the initial formulations (HGC and SDS capsule A) showed a very high inter-patient variability in terms of pharmacokinetics, leading to the development of SDS sachet formulation with more consistent PK profiles. Overall, BEZ235 treatment was well tolerated irrespective of the formulation and dose level. Most common tumor types included colorectal (~ 25%), breast (~ 20%) and lung (~ 10%) cancer. A significant number of patients had detectable reduction of the tumor load (minor tumor shrinkage < 30%) and/or prolonged disease stabilization (4 up to > 18 months), among them several with PI3K-pathway activated tumors. In addition, more than half of the patients showed reduction of [FDG]^18^ uptake on PET scan including several with partial metabolic response [35, 36].

Others and we have previously shown that BEZ235 has potent anti-leukemic activity in preclinical models of ALL and AML [10, 19, 20, 23]. This phase I study was conducted to determine the maximum tolerated dose (MTD) and recommended phase II dose of BEZ235 and to evaluate the safety, preliminary efficacy and pharmacokinetics of BEZ235 in patients with relapsed or refractory ALL or AML.

## Material and methods

### Patient selection

For dose escalation phase patients were included over 18 years of age with either relapsed or refractory AML after standard therapy in which conventional salvage therapy was inappropriate or who were previously untreated for the reason of age, poor prognosis, or concurrent medical conditions and therefore were considered unfit for standard induction therapy, or patients with T-ALL or Philadelphia chromosome negative BCP-ALL suffering from relapse (after at least induction and consolidation chemotherapy) or showing refractory disease and having no option to be treated with standard therapy, or patients with relapsed or refractory Philadelphia chromosome- or BCR-ABL1-positive BCP-ALL or CML-BP after first- and second-line treatment (including at least two tyrosine kinase inhibitors (TKIs)) or showing molecular relapse or molecular progression in the minimal residual disease (MRD) measurement including the detection of the T315I mutation or other mutations conferring resistance to available TKI treatment. MRD analysis was based on the detection of BCR-ABL1 transcripts by RT-PCR. These patients could not be eligible for allogeneic stem cell transplant (SCT) at the time of enrolment. In the presence of a T315I mutation, prior treatment with a second TKI was not required.

Additional eligibility criteria included serum bilirubin ≤1.5 x the institutional upper limit of normal (ULN) except with known Gilbert’s Syndrome, alanine transaminase (ALT) and aspartate transaminase (AST) ≤ 3 x the ULN (or ≤ 5.0 x ULN in case of hepatic infiltration by leukemia), INR ≤ 1.5, serum creatinine ≤2 mg/dl or creatinine clearance ≥50 ml/min, fasting plasma glucose (FPG) ≤ 160 mg/dL, HbA1c ≤ 8% and WBC ≤ 30 × 10^9^/L.

Cytoreductive treatment was allowed up to 1 week prior to first dosage for typical prephase or maintenance medication for ALL like vincristine, mercaptopurine, low-dose (≤15 mg/m^2^) MTX and low-dose cyclophosphamide (cumulative dose ≤1 g/m^2^) and treatment with steroids and hydroxyurea were allowed up to 1 day before first dosage.

Patients were excluded showing acute promyelocytic leukemia (APL), diabetes mellitus (insulin dependent or history of gestational diabetes), reduced cardiac function (left ventricular ejection fraction < 45% and/or QTcF > 480 msec on screening ECG) and patients with active ≥ grade II graft versus host disease (GVHD). Patients requiring treatment with medication having the potential to interact with BEZ235 in the respect of QT prolongation or metabolization via p450 cytochrome enzymes were also excluded.

The BID schedule investigated in this trial was selected based on preliminary data obtained from the solid tumor phase I study CBEZ235ZUS07T.

### Study design

This was a phase I, single center, open-label study designed to assess the safety, tolerability, preliminary efficacy and PK of BEZ235. During the initial dose escalation phase, successive patient cohorts were scheduled to receive BEZ235 orally twice a day during 28-day cycles. Dose escalation was based on a “rolling-six” design, a modification of the 3 + 3 design. The first cohort of subjects received a starting dose of 400 mg/BID. Dose escalation for subsequent patient cohort of subjects was guided by the incidence of BEZ235-related adverse events as graded by NCI Common Terminology Criteria for Adverse Events (CTCAE) v4.03 (http://evs.nci.nih.gov/ftp1/CTCAE) in the first 4 weeks of dosing (dose limiting toxicities (DLT) evaluation period). Up to 6 subjects could be enrolled to a dose level, although only 3 subjects were required to complete the DLT evaluation period prior to enrolling subjects in the next higher dose cohort. The patient population for the determination of MTD consisted of patients who fulfilled the minimum safety evaluation requirements of the study, which were met if in cycle 1, the patient has been treated with BEZ235 for ≥21 days, observed for ≥28 days following the first dose, and completed all relevant safety evaluations, or experienced a DLT during cycle 1. Patients who did not meet these minimum safety evaluation requirements were regarded as ineligible for the MTD determining population and were replaced.

### Toxicity and safety assessment

Adverse events data and serious adverse events were analyzed taking into account the NCI-CTCAE v4.03 and were presented in frequency tables by grade. Haematological and clinical biochemistry toxicities were assessed from laboratory test parameters. For patients with multiple occurrences of the same event the maximum grade (worst) was used. The safety analyses were performed in the safety population.

### Response evaluation

Assessment of response was first performed at the end of cycle 1 (day 29) and after every subsequent cycle until CR or CRi were achieved. After CR or CRi, ongoing response was evaluated on day 29 of every even cycle and at end of treatment.

In case of ALL CR was defined as: absence of circulating blast cells, Polymorphonuclear > 1000 /mm^3^, platelets > 100,000 /mm^3^, no extra medullary involvement and a normal bone marrow smear. In case of AML CR was defined as: < 5% leukemic blasts in bone marrow aspirate and absence of Auer rods, no leukemic blasts in peripheral blood, neutrophils ≥1.0 × 10^9^/L, platelets ≥100 × 10^9^/L and no evidence of extramedullary disease. CRi was defined as: < 5% leukemic blasts in bone marrow aspirate and absence of Auer rods, no leukemic blasts in Peripheral blood, neutrophils < 1.0 × 10^9^/L, platelets < 100 × 10^9^/L and no evidence of extramedullary disease.

### Pharmacokinetic analysis

Blood for Pharmacokinetic (PK) analysis was collected on day 1 of cycle 1 prior to and 2 h, 4 h, and 8 h after the first administration of BEZ235, after 24 h immediately preceding the second administration of BEZ235 and on day 15 of cycle 1 immediately prior to administration of BEZ235. All blood samples were taken by either direct vein puncture or an indwelling cannula in accordance with the assessment schedule (Table 1). If a patient had a central line, blood sampling was also obtained from this source. The sample tubes were to be labelled with pre-printed labels which contain the following information: protocol number, centre number, patient number, patient initials, sample number, date sample taken and actual time of sample. All samples were given a unique sample number and the exact clock time of dosing, as well as actual sample collection date and time will be captured on the CRF page. Sampling problems were noted in the comments field of the CRF page. All pharmacokinetic specimens were stored frozen at least at − 20 °C until shipment. Samples were packed carefully with suitable packing material and dry ice to keep them frozen during shipment. Laboratory analyses of specimens collected in this study for determination of drug or metabolite concentrations were conducted by the central laboratory at the University of Dresden.

**Table 1:** Blood collection plan

Sample (Blood)	Volume (mL)	Cycle	Day	Sample No.	Scheduled time relative to BEZ235 dosing post-dose (hours)
	3	1	1	1	Pre-dose
				2	1 h post-dose
	3	1	1	3	2 h post-dose
	3	1	1	4	3 h post-dose
				5	5 h post dose
	3	1	1	6	8–12 h post-dose
(optional)	3	1	2	7	24 h post-dose (trough)
	3	1	15	8	Pre-dose
	3	1	15	9	1 h post-dose
	3	1	15	10	3 h post-dose
	3	1	15	11	5 h post-dose
(optional)	3	1	15	12	8–12 h post-dose

Blood sample collection plan in the BEZ 235 phase I trial for PK and PD analysis is shown

Pharmacokinetic analyses were performed in 21 patients and in all over 235 plasma samples. BEZ235 plasma levels were determined by HPLC and fluorescence detection. For the HPLC assay zinc sulfate (5 g/100 ml) and acetone were added to plasma samples to precipitate protein. 300 μl plasma were combined with 200 μl ZnSo4 and 300 μl acetone, were vortexed for 5 min and subsequently centrifuged for 10 min followed by HPLC analysis. HPLC analysis was performed using a ZirChrom PBD column, which was equilibrated with 95% water, 1 ml TEMED, 5% methanol and adjusted to a pH of 4 with phosphoric acid (800 μl; pH 3.6 / l Eluent). For elution an MN 125–4, 5 μm, Rp SelectB column was used. The eluent was composed of 45% water, 1 ml TEMED and 55% methanol, adjusted to pH 2 with phosphoric acid (2250 μl pH 2/l Eluent). Detection was performed with a fluorescence detector at 270 nm extinction and 480 nm emission. This methodology was validated to a detection limit of 1 ng/ml BEZ235. The intra- und inter-assay variation for 10 samples each per measurement was below 10%. PK parameter analysis was performed for every patient individually based on a 2-compartment model using the TOPFIT PK computer program. For weighting the concentration was set to 1/y. All correlation coefficients were ≥ 0,85.

### Pharmacodynamic parameters

Pharmacodynamic (PD) analysis included assessment of phosphorylation of AKT, S6 and 4EBP1 by WB and flow cytometry. Primary patient samples adequate for Western blot PD and flow cytometry analysis were obtained from bone marrow and or peripheral blood prior to therapy and on days 1, 2, 15, 28 and/or end of treatment (EOT). Jurkat T-ALL cells were used as positive control in each case.

#### Minimal residual disease

MRD assessment was performed after each cycle of BEZ235. Disease specific MRD markers were used for each patient individually. All transplanted patients were monitored additionally via regular chimerism analysis.

### Mutation analysis

The presence of *PI3KCA*, *AKT* or *PTEN* mutations was evaluated by direct sequencing of exons with known mutation hotspots.

### Whole genome and RNA sequencing

Samples from representative time points during treatment with BEZ235 were used for whole genome and RNA sequencing in the best responding patient described below. Herein a skin punch biopsy was used as non-malignant tissue control and a bone marrow sample from relapse after allogeneic SCT before initiation of therapy with BEZ235 as tumor sample.

DNA from tumor (mononuclear bone marrow cells) and normal tissue (skin punch biopsy) were extracted using the DNeasy Blood & Tissue kit (Qiagen). Sequencing libraries were prepared using the TruSeq DNA Nano kit (Illumina) and sequenced on an Illumina HiSeq X sequencer in paired and mode with a read length of 150 bp.

For analysis of WGS data the OneTouch Pipeline (OTP) developed at the German Cancer Research Center (DKFZ) has been employed. Details of the pipeline as described in Reisinger E et al. [37] In brief, raw reads were aligned using bwa mem and SNVs were called using samtools (mpileup, bcftools) and platypus. Indels were called with platypus. The used alignment, SNV- and InDel-calling are based on the established workflows in the International Cancer Genome Consortium (ICGC) and are basically described in Alioto et al. [38] Structural variants were called with SOPHIA (https://bitbucket.org/utoprak/sophia/src/master/) and CNVs were determined using ACESeq (https://www.biorxiv.org/content/10.1101/210807v1.full). The WGS pipeline of the OTP has been extensively validated and used for WGS data analysis in multiple international (ICGC) as well as national (MASTER, INFORM) cancer sequencing programs.

Total RNA was extracted from mononuclear bone marrow cells using the RNeasy Mini kit (Qiagen). Sequencing libraries were prepared using the TruSeq Stranded Total RNA kit (Illumina) and sequenced on an Illumina HiSeq 4000 in paired end mode with a read length of 100 bp. RNA sequencing data was analyzed using STAR [37, 39] and fusion transcripts were detected using STAR-Fusion (https://www.biorxiv.org/content/early/2017/03/24/120295).

### Statistical considerations

This was a phase I multiple ascending dose study in which a following variant of the 3 + 3 rule (“Rolling Six”), a minimum of 6 and a maximum of 12 patients was recruited for the dose escalation and another 5 were planned for the expansion phase in ALL/AML. Demographics and baseline Characteristics: standard descriptive statistics, such as the mean, median, range and proportion, were used to summarize the patient sample and to estimate parameters of interest. Ninety-five per cent confidence intervals were provided for estimates of interest where possible. Statistical tests for comparison of different patient cohorts were not feasible due to low patient number.

### Safety population

All patients who received at least one dose of study medication and had at least one post-baseline safety assessment (as evaluated by the existence of at least one adverse event CRF, including the case where no adverse event is reported) were included.

### MTD determining population

The MTD determining population enclosed all patients from the safety population who either received enough treatment and had sufficient safety evaluations or discontinued due to unacceptable toxicity. The minimum safety evaluation requirements were met if, in cycle 1, the patient has been treated with BEZ235 for ≥21 doses. In addition, the patient must have been observed for ≥28 days following the first dose, and must have completed sufficient safety evaluations or the patient experiences DLT during cycle 1. Patients who did not meet these minimum safety evaluation requirements were regarded as ineligible for the MTD determining population and were replaced.

### Trial registration

ClinicalTrials.gov, identifier NCT01756118, registered 19th December 2012, https://clinicaltrials.gov/ct2/show/NCT01756118.

## Results

Twenty-four patients with relapsed or refractory AML (*n* = 12), BCP-ALL (*n* = 10), T-ALL (*n* = 1) and CML-BP (*n* = 1) were enrolled between 21-Jun-2012 and 25-Nov-2013. Five patients were in first and 5 patients in second or later relapse, 14 patients were refractory or in refractory relapse. Sixteen patients had recurrent disease after allogeneic SCT. Extramedullary leukemia was present in 5 patients. Baseline patient demographics and disease characteristics are provided in Table 2.

**Table 2:** Baseline patient characteristics

Patient characteristics	n (%)
Age	
Median (years)	61
Range (years)	29–82
Sex	
male/female	15 (63) / 9 (38)
Disease	
ALL	11 (46)
BCP-ALL	10
T-ALL	1
AML	12 (50)
CML-BP	1 (4)
Extramedullary disease	5 (21)
SCT prior to study therapy	16 (67)
SCT in AML patients	6
SCT in ALL patients	9
SCT in CML-BP patient	1

Baseline patient characteristics reveal a median age of 61 and mainly patients with BCP-ALL and AML, who previously in most cases underwent an allogeneic SCT

### Treatment and adverse events

Six patients were evaluated at the starting dose of 400 mg BID. The most frequent non-hematologic adverse events (AEs) were gastrointestinal primarily of grade 1 and 2 with diarrhea (*n* = 15), nausea/vomiting (*n* = 19), stomatitis/mucositis (*n* = 16), liver function test increase (*n* = 11), Anorexia (*n* = 8) and hyperglycemia (*n* = 6). Grade 3/4 AEs included diarrhea (*n* = 2), liver function test increase (*n* = 2), hyperglycemia (*n* = 2), lipase increase (*n* = 1), amylase increase (*n* = 1), gastroenteritis (*n* = 1) and esophagitis (*n* = 1). The hematologic side effects included neutropenia (*n* = 5 with *n* = 2 grade 3/4), anemia (*n* = 4 with *n* = 2 grade 3/4) and platelets count decrease (*n* = 4 with all grade 3/4) (see Table 3).

**Table 3:** Treatment emergent adverse events

	400 mg (*n* = 6)				300 mg (*n* = 18)				All patients (*N* = 24)			
	All grades		G 3/4		All grades		G 3/4		All grades		G 3/4	
	n	(%)	n	(%)	n	(%)	n	(%)	n	(%)	n	(%)
Nausea,Vomiting	6	100	0	0	11	61	0	0	19	79	0	0
Diarrhea	6	100	2	33	11	61	0	0	17	71	2	8
Mucositis oral	6	100	1	17	11	61	0	0	17	71	1	4
Liver function tests increased	1	0	1	0	10	56	1	6	11	46	2	8
Anorexia	5	83	0	0	3	17	0	0	8	33	0	0
Hyperglycemia	1	17	1	17	7	39	1	6	8	33	2	8
Dyspepsia	2	33	0	0	3	17	0	0	5	21	0	0
Stomach or abdominal pain	2	33	0	0	3	17	0	0	5	21	0	0
Neutropenia	1	17	1	17	4	22	1	6	5	21	2	8
Anemia	1	17	0	0	3	17	2	11	4	17	2	8
Fatigue	2	33	0	0	2	11	0	0	4	17	0	0
Platelet count decreased	0	0	0	0	4	22	4	22	4	17	4	17
Bloating, Flatulence	2	33	0	0	2	11	0	0	4	17	0	0
Anal pain	3	50	0	0	0	0	0	0	3	13	0	0
C-peptide increase	0	0	0	0	3	17	0	0	3	13	0	0
Constipation	2	33	0	0	0	0	0	0	2	8	0	0
Alkaline phosphatase increased	0	0	0	0	2	11	0	0	2	8	0	0
Lipase increased	0	0	0	0	2	11	1	6	2	8	1	4
Weight loss	2	33	0	0	0	0	0	0	2	8	0	0
Hypertriglyceridemia	2	33	0	0	0	0	0	0	2	8	0	0
Hoarseness	2	33	0	0	0	0	0	0	2	8	0	0
Colitis	0	0	0	0	1	6	0	0	1	4	0	0
Gastroenteritis	0	0	0	0	1	6	1	6	1	4	1	4
Esophagitis	0	0	0	0	1	6	1	6	1	4	1	4
Erythema multiforme	1	17	0	0	0	0	0	0	1	4	0	0
Skin infection	1	17	0	0	0	0	0	0	1	4	0	0
Nail ridging	0	0	0	0	1	6	0	0	1	4	0	0
Urea increased	0	0	0	0	1	6	0	0	1	4	0	0
Serum amylase increased	0	0	0	0	1	6	1	6	1	4	1	4
Dry skin	0	0	0	0	1	6	0	0	1	4	0	0
Insomnia	0	0	0	0	1	6	0	0	1	4	0	0
Muscle cramps	1	17	0	0	0	0	0	0	1	4	0	0
Dizziness	1	17	0	0	0	0	0	0	1	4	0	0
Dysgeusia	1	17	0	0	0	0	0	0	1	4	0	0
Alopecia	1	17	0	0	0	0	0	0	1	4	0	0

Treatment associated adverse events are listed according to frequency. Gastrointestinal AEs like vomiting, diarrhea and oral mucositis were most frequent but only in rare cases with higher grades (3/4). The most frequent AE with higher grade was lowering of thrombocyte count

400 mg BID was considered not tolerable for prolonged administration and 18 pts. were subsequently treated at dose level − 1 (300 mg BID). The most common non hematologic side effects especially the gastrointestinal related AEs showed a clear dose dependency as occurrence under 400 mg BEZ235 BID vs. 300 mg BID demonstrates (see Table 3): nausea/vomiting, diarrhea and mucositis (all 3: 100% vs. 61%), anorexia (83% vs. 17%), dyspepsia and stomach and abdominal pain (33% vs. 17%), fatigue, bloating and flatulence (33% vs. 11%), anal pain (50% vs. 0%), constipation, weight loss and hypertriglyceridemia (33% vs. 0%) and erythema multiforme, skin infection muscle cramps, dizziness, dysgeusia and alopecia (17% vs. 0%). Other AEs especially in terms of hematologic toxicity showed an inverse or unclear dose dependency: liver function test increase (17% vs. 56%), neutropenia (17% vs. 22%), anemia (17% vs. 17%), platelet count decrease (0% vs. 22%), C-peptide increase (0% vs. 17%), alkaline phosphatase increase, lipase increase (0% vs. 11%), colitis, gastroenteritis, esophagitis, nail ridging, urea increase, serum amylase increase, dry skin and insomnia (0% vs. 6%). The same dose dependency also holds true in grade 3/4 AEs regarding gastrointestinal toxicity: diarrhea (33% vs. 0%), mucositis oral (17% vs. 0%), hyperglycemia (17% vs. 6%) but also in neutropenia (17% vs. 6%). Inverse or no correlation in grade 3/4 was given in liver function tests increase (0% vs. 6%), anemia (0% vs. 11%), platelet count decrease (0% vs. 22%) and lipase increase, serum amylase increase, gastroenteritis and esophagitis (0% vs. 6%) (see Table 4). No patient starting at dose level − 1 was dose-reduced and none discontinued BEZ235 because of toxicity, 300 mg BID was selected as the RP2D.

**Table 4:** Adverse events grade 3/4 with dosage correlation

	400 mg (*n* = 6)		300 mg (*n* = 18)		All patients (*N* = 24)	
	Grade 3/4		Grade 3/4		Grade 3/4	
	n	(%)	n	(%)	n	(%)
Diarrhea	2	33	0	0	2	8
Mucositis oral	1	17	0	0	1	4
Neutropenia (Neutrophil count decreased and/or WBC decreased)	1	17	1	6	2	8
Liver function test: values increased	1	17	1	6	2	8
Gastroenteritis	0	0	1	6	1	4
Esophagitis	0	0	1	6	1	4
Serum amylase increased	0	0	1	6	1	4
Lipase increased	0	0	1	6	1	4
Anemia	0	0	2	11	2	8
Platelet count decreased	0	0	4	22	4	17

Grade 3/4 AEs showed no clear dose correlation but predominance of gastrointestinal AEs in the 400 mg BIS cohort and a higher heamatotoxicity rate in the 300 mg BID cohort

### Dose-limiting toxicities and RP2D

In the starting cohort (dose level 400 BID), four patients completed cycle 1, i.e. the 4 week time period for assessment of DLT. While no formal DLT were observed, tolerability was poor, with stomatitis and a wide range of gastrointestinal toxicities which required treatment interruptions in 3 of 6 patients. Treatment at the 400 BID dose level would not be possible beyond 4 weeks and would be tolerated for only less than 4 weeks in a substantial proportion of patients. At the 300 mg BID dose level tolerability was far better with no DLT and no adverse events that were considered incompatible with continued BEZ235 administration.

In conclusion, the RP2D for BEZ235 was determined to be 300 mg BID, without formal definition of DLTs and an MTD.

### MRD monitoring

Individual MRD marker establishment leaded to the following results: in the CML-BP and the Ph + BCP-ALL patient *BCR-ABL1* (p210 and p190 transcript respectively) analysis via real time PCR including sanger sequencing for mutational analysis were used. Mutational analysis revealed no mutations in the Ph + BCP-ALL patient and a E255K and G250E mutation in the BP-CML patient. The 9 other Ph- BCP-ALL patients were monitored via individually established Ig rearrangement markers using real time PCR methods. In one BCP-ALL patient we additionally analyzed the MLL-ENL transcript. One T-ALL patients was monitored via TCR-rearrangement. The 12 AML patients were monitored via FLT3 ITD (*n* = 2), MLL-PTD (*n* = 1), CEBPA (*n* = 1), JAK2 (*n* = 1) and NPM1(*n* = 1). In *n* = 6 AML patients no MRD marker was established.

### Efficacy

Clear Responses were observed in 3 of 24 patients, all of them ALL (3/11). One patient with pro-B ALL reached sustained complete remission (CR). One patient with T-ALL showed a reduction in blast count in peripheral blood from 64 to 5%. One patient with *BCR-ABL1* positive BCP-ALL showed a reduction of bone marrow blasts from 70 to 16%. Notably one patient with AML harboring the *MLL (KMT2A)* aberration remained stable for 4 months but was not counted as a responder.

**Fig. 1:**
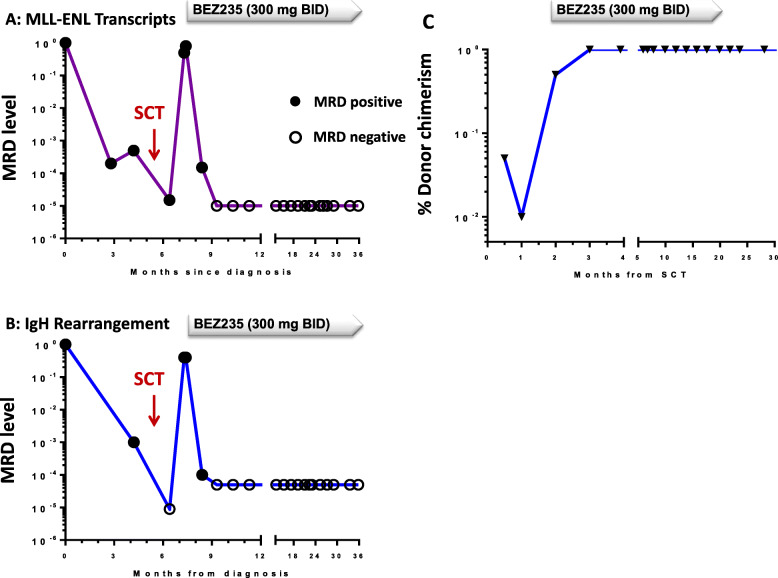
*MRD markers and donor chimerism in best responding patient.* **a/b** MRD measurement via *KMT2A/MLLT1* transcripts and IgH rearrangement in the best responding BPC-ALL patient revealed a response via SCT, but finally sustaining molecular response was reached via BEZ 235 treatment. **c** Donor chimerism in bone marrow was incomplete after allogeneic SCT with 1–5% autologous signal at day + 29 after SCT and dropping at early relapse. Soon after start of treatment with BEZ complete chimerism was reached

The patient who achieved a CR is particularly noteworthy in view of the depth and sustained nature of the response. This was a female patient first diagnosed with a pro-B ALL carrying the t(11;19) translocation and molecular analysis showing a *KMT2A/MLLT1* fusion transcript. The patient had received induction and consolidation therapy according to the GMALL protocol GMALL 07/03 and underwent allogeneic SCT in CR1 with lack of molecular remission with TBI-based MAC SCT and a matched unrelated donor. Relapse occurred 6 weeks after SCT, and the patient was enrolled in this study and allocated to the 300 mg BID cohort. Complete hematologic remission was documented 28 days after starting BEZ235. Assessment of MRD by RT-PCR for *KMT2A/MLLT1* transcripts and immunoglobulin gene rearrangement analysis demonstrated complete molecular response 6 years after starting BEZ235 and in parallel donor chimerism increased from 40 to 60% autologous signal to complete chimerism after 28 days and has remained complete to date. RT-PCR analysis, results of Immunoglobulin rearrangement and donor chimerism are depicted in Fig. 1a-c. Disease progression lead to discontinuation in 22 of 24 pts. with a median time to progression of 27 days (4 days–112 days).

ALL patients showed the clearest single-agent anti leukemic effect resulting in an overall response rate of 27% in these patients and one patient showing an ongoing molecular remission. The response showed no correlation with the assessment of PK and PD markers of PI3K signaling.

### Pharmacokinetic analysis

**Fig. 2:**
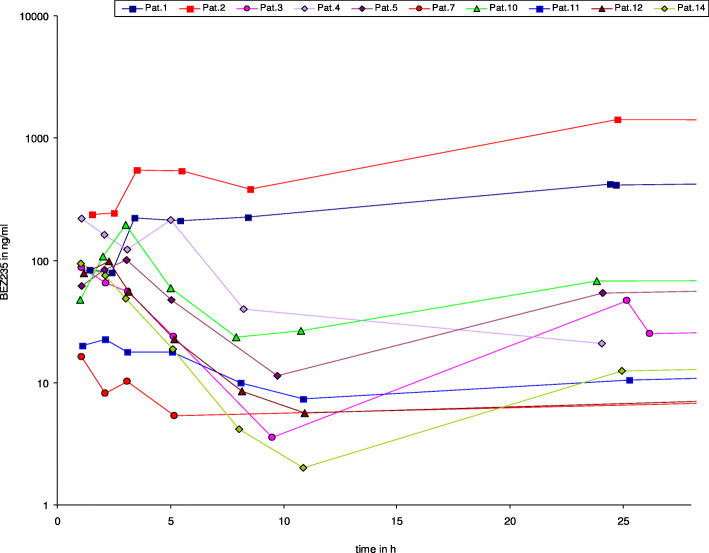
*Plasma concentration BEZ235 day 1*. Plasma concentrations of BEZ 235 at day 1 showing a broad interpatient variability. BEZ 235 concentrations did not correlate with response to treatment

**Fig. 3:**
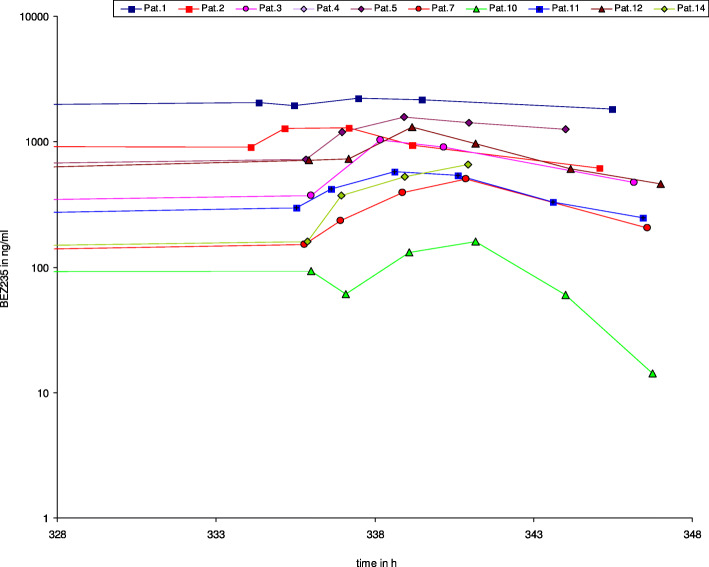
*Plasma concentration BEZ235 day 15.* Plasma concentrations of BEZ 235 at day 15 showing again a broad interpatient variability. BEZ 235 concentrations did not correlate with response to treatment

PK analysis revealed a remarkably high inter-individual variability of the AUC and Cmax, which covered a range from 5 to 1000 ng/ml. This variability is depicted in Fig. 2 for day 1 and persisted on day 15 at steady state (Fig. 3), with no dose-dependency. We observed non-linear PK when comparing day 1 and 15 data, with a proportionally too high AUC and Cmax at day 15. These PK data are in line with preliminary data generated in a separate study of BEZ235 for solid tumors using a once daily dosing schedule. Our PK data are consistent with a saturation effect in the elimination of BEZ235. Identification of 2 or 3 additional peaks in all plasma samples most likely representing metabolites of BEZ235 also suggests saturation of a metabolic pathway. Surprisingly, all three responders in whom PK data are available had low steady state trough levels below 100 ng/ml.

### Mutations and PD parameters

No activating mutations of *PIK3CA*, *AKT* or *PTEN* were identified in any of the 24 pts. Results of the PD analyses (phospho-flow cytometry and western blot (WB)) analysis showed no signs of activation in any patient in AKT, S6 and 4EBP1.

### Genetic characterization of the tumor from the best responding patient

To gain additional insights into the mechanisms leading to prolonged response in one ALL patients we performed whole genome sequencing of tumor and normal tissue at a coverage of 80× and 40×, respectively. The germline control DNA was isolated from a skin biopsy and was contaminated with SCT donor DNA that hampered the analysis. We identified a large number of low allele frequency SNPs (AF < 0.1) that likely result from the contamination and are not real somatic SNPs. No functionally relevant somatic SNPs with an AF above 0.1 could be identified. The analysis also did not reveal functionally relevant SNVs in genes involved in PI3K/AKT/mTOR signalling pathway. RNA sequencing was primarily performed to identify gene fusions. These analyses confirmed the presence of the *KMT2A-MLLT1* that was previously identified in routine cytogenetics. Due to the nature of the tumor material and the lack of reference material differential gene expression analysis was not performed.

## Discussion

This study represents the first step on a development path that aims at improving cure rates for advanced hematologic malignancies including ALL, AML and CML-BP by simultaneously targeting different components of the PI3K/Akt/mTOR signaling pathway. PI3K/AKT/mTOR signaling is known to play a central role in up regulating cell proliferation, survival, and drug resistance in numerous hematologic malignancies. In this trial clinical response was observed in 16% of patients with 30% response in ALL patients. In this respect 3 patients with BCP-ALL showed remarkable responses: two patients showed hematologic improvement and one patient reached an outstanding long-lasting molecular remission with reconstitution of full donor chimerism. In AML cases, in 91% of patients disease progression lead to study discontinuation, but one patient showed stable disease for 4 months, which leads to the conclusion, that single-agent anti leukemic effect was most obvious in cases of ALL. Within these patients and especially within the responding cases phospho-flow and western blotting analysis revealed no evidence of PI3K pathway activation. Taking these results together and taking into account our inability to show activation of the PI3K/AKT/mTOR pathway in the baseline setting, the described analysis of PK and PD parameters necessarily did not correlate with response.

The described saturation effect in elimination of BEZ revealed in in the PK analysis may limit elimination and could conceivably contribute to the PK variability, but it remains to be determined whether this saturation effect, which was observed in most patients, is linked with the occurrence of adverse events. Further analysis of these proposed metabolites has not been possible to date.

Genetic analysis of the tumor sample from the best responding patient did not yield functionally relevant SNVs in genes involved in PI3K/AKT/mTOR signalling cascade. The mechanism underlying the prolonged remission observed in one patient with an exceptional response therefore remains unknown but it is possible that this patient belongs to a small subgroup of AML and ALL patients in which the PI3K pathway functions as oncogenic driver at the level of LSCs. Nevertheless, inhibition of one signaling component may not show a sufficient antitumor effect in most of the patients. The limited efficacy of single inhibitors in this pathway might be explained by the existence of signaling feedback loops mediated by p70S6K and PI3K. The effect of dual catalytic PI3K/mTOR inhibitors such as BEZ235 are supposed to overcome PI3K/Akt pathway reactivation, but in in fact the majority of patients in this trial showed no sufficient response.

The mechanisms involved in activation of this pathway have been examined in AML and ALL and include activating mutations of the FLT3 and the KIT tyrosine kinase receptors, NRAS or KRAS mutations, PI3K overexpression [40], low levels of PP2A phosphatase activity and autocrine/paracrine secretion of growth factors such as IGF-1 and VEGF [23]. The contribution of PTEN inactivation as a mechanism of PI3K pathway activation in AML is controversial; although PTEN is deleted in many solid cancers and T-cell acute lymphoblastic leukemia, PTEN deletion is extremely rare in AML [41–43]. To date it is known in T-ALL pathogenesis that PI3Kgamma or PI3Kdelta without presence of PTEN phosphatase tumor suppressor function can promote leukemogenesis, whereas loss of function of both isoforms inhibited tumor formation. The potential of a dual inhibitor to lower disease burden and prolong survival in mice and the fact that dual inhibitors limit proliferation and enhance proapoptotic pathways in human tumors further demonstrate the dependence of *PTEN* null mutation T-ALL on the combined activities of PI3Kgamma/delta. All these findings encourage the development of combined PI3Kgamma/delta inhibition as therapeutic strategy for T-ALL [28, 29]. The described mechanisms in the PI3K/Akt pathway may explain that response in AML as discussed was nearly not seen in this trial.

But according to preclinical data anti-tumor efficacy of BEZ is suggested in acute leukemia and in addition, preclinical and clinical data that demonstrate the relevance of mTOR and the anti-tumor effects of mTOR inhibition in numerous malignancies also suggest that compounds able to inhibit the PI3 kinase pathway at several levels simultaneously may possess greater anti-leukemic efficacy and be able to prevent development of drug resistance. Even though the clinical development of BEZ235 has been terminated because of suboptimal pharmacokinetic properties, dual inhibition of PI3 kinase and both mTOR complexes (C1 and C2) by molecules such as BEZ235 remains an attractive therapeutic approach for advanced malignant diseases. While therapeutic targeting of the PI3K/Akt/mTOR signaling cascade at multiple molecular levels has been shown preclinically to provide better antitumor effects than selective inhibition of only individual components of this pathway, a priori identification of leukemias likely to respond has so far proven elusive. In AML, constitutive PI3K-Akt-mTOR activation was shown to differ between patients with high constitutive pathway activation [44, 45]. Being associated with less monocytic differentiation and increased frequency of adverse karyotypes, indicating that activation level may depend on complex phenotypic differences [44, 45]. In B cell precursor ALL, we previously showed that combined suppression of PI3K, mTORC1 and mTORC2 displayed greater antileukemic activity than selective inhibitors of PI3K, mTORC1 or mTORC1 and mTORC2 irrespective of their genetic subtype and in case of *BCR-ABL1* positive ALL their responsiveness to ABL-directed kinase inhibitors [46]. In T-ALL, a vital role in Notch-driven thymocyte differentiation and leukemia has been assigned to mTOR complex 2, suggesting a potential role of mTOR C2 inhibition in undifferentiated T-ALL [47, 48]; however, activation of the PI3K axis is a common feature among T-ALL independent of differentiation stage.

Overall, the mechanisms by which the PI3K pathway is aberrantly activated, the relative contribution of different components of this pathway (PI3K, AKT, mTORC1 and mTORC2, S6 kinase etc.), positive and negative feedback mechanisms and the crosstalk with other signaling pathways differ substantially between tumor entities and between patients and cannot be assigned to specific subtypes. Accordingly, future treatment concepts involving inhibitors of PI3, AKT and mTOR may have to utilize different combination partners depending on the precise pathophysiological basis of PI3-K signaling in different diseases.

## Conclusions

The combined inhibition of PI3K and mTOR by BEZ235 interferes with a clinically meaningful driver pathway in a small subset of patients with ALL, with no benefit in patients with AML. Herein PK analysis and PD markers assessment associated with PI3K signaling did not correlate with response. The safety profile revealed a mainly gastrointestinal toxicity (apart from hematologic side effects) and the RP2D for BEZ235 was determined to be 300 mg BID, without formal definition of DLTs and an MTD. Taken these findings together we believe that the efficacy observed in the treated ALL patients warrants a further clinical exploration of dual PI3K/mTOR inhibition potentially as combination therapy with other small molecules or chemotherapeutics. Patients with Ph + BCP-ALL or T-ALL may benefit from these combination therapies and correct dosing can likely circumvent the toxicities observed in this trial.

## Data Availability

The datasets used and/or analyzed during the current study are available from the corresponding author on reasonable request.

## Abbreviations

AEs: Adverse events
ALL: Acute lymphoblastic leukemia
ALT: Alanine transaminase
AML: Acute myeloid leukemia
AST: Aspartate transaminase
BCP-ALL: B-cell precursor acute lymphoblastic leukemia
CML-BP: Chronic myeloid leukemia in blast phase
CNV: Copy number variation
DLT: Dose limiting toxicities
FLT3: Fms-like tyrosine kinase 3
FPG: Fasting plasma glucose
GVHD: Graft versus host disease
ICGC: International cancer genome consortium
LSCs: Leukemia stem cells
MRD: Minimal residual disease
MTD: Maximum tolerated dose
mTOR: Mammalian target of rapamycin
NCI-CTCAE: NCI common terminology criteria for adverse events
NOD/SCID: Non-obese diabetic/severe combined immunodeficiency mice
OTP: One touch pipeline
PD: Pharmacodynamic
Ph+: Philadelphia chromosome positive
Ph-: Philadelphia chromosome negative
PI3K: Phosphatidylinositol 3-kinase
PK: Pharmacokinetic
PTEN: Phosphatase and tensin homolog deleted on chromosome 10
RP2D: Recommended phase II dose
RTKs: Receptor tyrosine kinases
SCT: Allogeneic stem cell transplant
SV: Structural variation
T-ALL: T-cell acute lymphoblastic leukemia
TKIs: Tyrosine kinase inhibitors
ULN: Upper limit of normal
WB: Western blot
